# Urine collection using a 3-way urethral catheter for PuO_2_

**DOI:** 10.1007/s00540-023-03276-3

**Published:** 2023-10-31

**Authors:** Takao Kato, Yuki Shiko, Kaoru Koyama

**Affiliations:** 1 Department of Anesthesiology, Saitama Medical Center, Saitama Medical University, Kawagoe, Japan; 2 Biostatistics Section, Clinical Research Center, Chiba University Hospital, Chiba, Japan

**Keywords:** Urine oxygen tension, Acute kidney injury, Urine carbon dioxide tension

To the Editor:

Perioperative acute kidney injury (AKI) increases perioperative mortality and worsens long-term survival [1]. Hypoxia in the renal medulla is associated with development of AKI [2, 3]. Renal medullary tissue oxygen partial pressure correlates with urine oxygen tension (PuO_2_) in the bladder, and PuO_2_ measurement may be useful for early diagnosis of AKI [4–7]. The validity of PuO_2_ was discussed in a 2021 Editorial in *Anesthesiology* [8].

We have developed an intermittent PuO_2_ monitoring method using a blood gas analyzer, and found that PuO_2_ < 130 mmHg at 6 h and < 88.6 mmHg at 12 h after ICU admission in cardiac surgery using cardiopulmonary bypass (CPB) were predictive of onset of AKI (AUC = 0.68, 0.64), allowing earlier diagnosis than that with Kidney Disease Improving Global Outcomes diagnostic criteria using creatinine and urine output [9] and without requiring specialized equipment. However, one problem was the proximal position of the urine collection port, which resulted in high PuO_2_ due to contact of urine with air or oxygenation from the bladder wall. Previous studies have found PuO_2_ of 89 ± 22 mmHg (mean ± SD) [6] and 65.5 ± 34.5 mmHg [7], while PuO_2_ was 120 ± 30 mmHg in our preliminary study [9], which may explain the low AUC. In addition, it has not been possible to collect urine at the necessary time during surgery (especially during CPB) because the required puncture through a urine collection port interfered with surgical procedures in our preliminary study [9].

To address these problems, we examined use of a 3-way urinary catheter (3-way Silicone Foley Catheter, Fuji Systems, Japan) to improve the accuracy of PuO_2_ measurements with a blood gas analyzer (ethics approval number: sou 2021-122). This urinary catheter has a tip lumen modified from a one-hole to two-hole type, which is advantageous for urine aspiration. In this study, we examined collection of urine intermittently and anaerobically directly from the bladder using this 3-way catheter, and compared the measured PuO_2_ with our preliminary results. Verification using toy balloons and plastic containers confirmed that liquid could be aspirated from the tip lumen of the 3-way catheter, but this has not been verified in vivo.

To ensure consistent and standard urine collection, a 120-cm pressure-resistant extension tube was connected to the tip lumen. Subsequently, 4 ml of urine was aspirated and disposed of using a plastic syringe, followed by collection of 1 ml of urine using a 1 ml plastic syringe. The collected urine sample was immediately subjected to measurement with a blood gas analyzer. In cases in which aspiration was not successful, the urethral catheter was temporarily clamped for 10 min, after which urine collection was attempted once more. The planned number of subjects was 12 to account for possible failure of urine collection, with enrollment terminated if urine was not collectable at > 50% of measurement times in the first 6 cases. After 6 patients, the study was terminated because collection of urine was only possible in one patient, and only after admission to the ICU (8 points). No patient developed AKI.

PuO_2_ was 75 ± 12 mmHg and urine carbon dioxide tension (PuCO_2_) was 55 ± 3.1 mmHg, compared to 45 ± 12 mmHg in our preliminary study [9]. Fick’s law suggests that when urine reaches equilibrium with the atmosphere, PuO_2_ approaches 160 mmHg and PuCO_2_ 0 mmHg. If the urine is in contact with the bladder wall, PuO_2_ and PuCO_2_ are expected to approach the values of partial pressure of arterial blood oxygen (PaO_2_) and partial pressure of arterial blood carbon dioxide (PaCO_2_), respectively. The mean PuO_2_ in a patient without AKI was lower than in our earlier study including patients with AKI, while PuCO_2_ was higher. These results suggest that urine collection may have been more anaerobic in the current study. Silverton et al. used a urine output > 0.5 ml/kg/h for > 30% time periods as a criterion for inclusion [10], but PuCO_2_ may be a more objective indicator, as a previously unrecognized finding, even in continuous PuO_2_ measurement using fiber-optic probes.

In the 6 cases, saline infusion could be performed even when urine could not be aspirated from the tip lumen, and urine output was maintained at > 0.5 ml/kg/h. To lower the aspiration pressure, we tried suctioning gently and removing the extension tube, but urine could not be aspirated. Therefore, we hypothesized that both holes at the tip of the urinary catheter were in contact with the bladder wall. It may have been helpful to use ultrasonography to confirm the bladder contents and determine the cause of the failure to aspirate urine.

This preliminary study suggests that urine collection from the tip of the urethral catheter may increase the accuracy of PuO_2_ and that PuCO_2_ measurements may reflect the accuracy of PuO_2_ measurements. The current 3-way urethral catheter tip lumen is considerably less likely to be able to collect urine from within the bladder in vivo. It is important to collect urine from close to the tip of the main lumen, where the urine originally drains out. An improved device to allow urine collection could enable intermittent measurement of urine oxygen tension as a real-time AKI biomarker using a blood gas analyzer.
